# Active microorganisms and potential metabolic pathways mediating anaerobic degradation of DMSP in anoxic saltmarsh sediment

**DOI:** 10.1093/ismeco/ycaf180

**Published:** 2025-09-09

**Authors:** Susan E G Hawthorne, Stephania L Tsola, Ornella Carrión, Jonathan D Todd, Özge Eyice

**Affiliations:** School of Biological and Behavioural Sciences, Queen Mary University of London, London, United Kingdom; School of Biological and Behavioural Sciences, Queen Mary University of London, London, United Kingdom; School of Biological Sciences, University of East Anglia, Norwich, United Kingdom; School of Biological Sciences, University of East Anglia, Norwich, United Kingdom; School of Biological and Behavioural Sciences, Queen Mary University of London, London, United Kingdom; School of Biosciences, University of Birmingham, Birmingham, United Kingdom

**Keywords:** DMSP, DMS, methane, saltmarsh, stable-isotope probing, metagenomics

## Abstract

Dimethylsulfoniopropionate (DMSP) is a globally abundant organosulfur compound produced by marine organisms, where it plays key physiological roles in stress protection and serves as a major source of carbon, sulfur, and energy for microbial communities. Importantly, DMSP degradation contributes to the formation of the climate-active gas dimethyl sulfide (DMS), which can drive the production of potent greenhouse gases, methane and carbon dioxide, in anoxic environments. While aerobic DMSP degradation is well studied, its fate under anoxic conditions remains poorly understood, and the microbial populations and metabolic pathways underlying these biotransformations are virtually unknown. Here, we present the first detailed investigation of microbial DMSP cycling in anoxic saltmarsh sediments. Our sediment samples had high in situ DMSP concentrations (up to 7.7 μmol/g) and the conversion efficiencies of DMSP to DMS under anoxic conditions (~68%) were comparable to those in oxic environments. Furthermore, using ^13^C-labelled DMSP in stable isotope probing (SIP) experiments, combined with 16S rRNA gene sequencing and metagenomics, we identified *Amphritea* (*Oceanospirillales*) as a key active DMSP degrader, likely operating via the *dddD*-encoded lysis pathway. Additional taxa, including *Geopsychrobacter*, were implicated as potential secondary consumers, while *Arcobacteraceae* may contribute to sulfur cycling rather than direct DMSP catabolism. This study uncovers a previously overlooked route for DMSP transformation via anaerobic metabolism, expands the known metabolic roles of saltmarsh microorganisms and highlights the potential for DMSP to drive climate-active gas production in anoxic coastal ecosystems.

## Introduction

Many algae, bacteria, corals and plants produce billions of tons of the tertiary sulfonium compound, dimethylsulfoniopropionate (DMSP), each year in marine ecosystems [1]. DMSP serves multiple functions including protection against oxidative stress, temperature fluctuations, osmotic and hydrostatic pressure and as a cue in chemotaxis [1, 2]. Furthermore, it is a major source of carbon, sulfur and energy for marine microbes through its catabolism, which can yield climate-active gases dimethyl sulfide (DMS), methanethiol (MeSH) and methane with prominent impacts on climate [3, 4]. For instance, every year 19 to 50 teragrams of DMS are released into the atmosphere, where its oxidation products form cloud condensation nuclei that exert a negative radiative forcing comparable to the warming effect of anthropogenic carbon dioxide (CO_2_) [5, 6].

**Figure 1:**
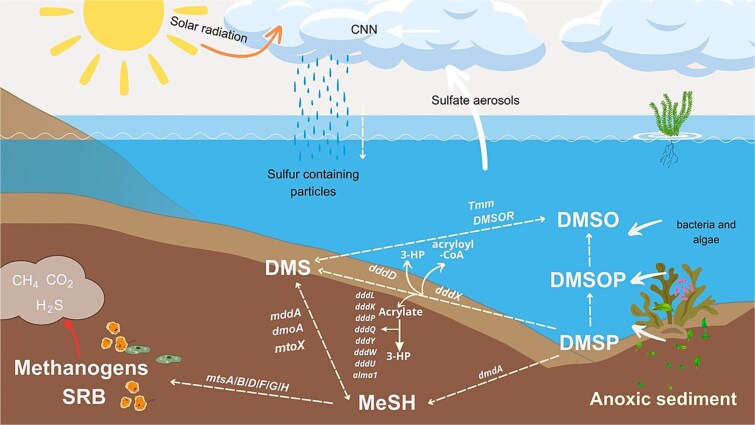
Metabolic routes to dimethylsulfoniopropionate (DMSP) catabolism. The demethylation pathway leads to the production of methylmercaptopropionate (MMPA) while the cleavage pathway results in the liberation of dimethylsulfide (DMS). The *dddD* gene results in additional production of 3-HP while the other series of *ddd* genes results in the additional production of acrylate, which may further be broken down into 3-HP. the *dddX* gene breaks down DMSP via a two-step reaction producing DMS and acryloyl-CoA. DMS is further metabolised by methanogens and sulfate-reducing bacteria (SRB), producing global warming gases methane (CH_4_) and CO_2_ in anoxic sediments. CCN: Cloud condensation nuclei; MeSH: Methanethiol; DMSOP: Dimethylsulfoxonium propionate; DMSO: Dimethylsulfoxide.

Mechanistically, DMSP is cleaved via intracellular DMSP-lyase enzymes (*ddd+*) in algae and bacteria to produce DMS and acrylate or 3-hydroxypropionate (3-HP; Fig. 1) [7–11]. An alternative pathway involves the demethylation of DMSP to methylmercaptopropionate (MMPA) via the *dmd* gene [12]. Many bacteria possess the genetic machinery for both pathways and may switch between them depending on environmental conditions such as oxidative stress, UV radiation, temperature and nutrient availability [13].

Due to the global significance of DMSP, substantial research efforts have been dedicated to understanding its production and degradation in aerobic marine waters and sediments. However, high concentrations (91–128 nmol/g) of DMSP were also detected in surface saltmarsh sediments [14–15]. In our previous study, we measured DMSP concentrations in anoxic saltmarsh sediments and reported levels of up to approximately 10 nmol/g [15]. More recently, Tebbe et al. detected even higher concentrations (up to around 67 nmol/g) in the anoxic layers of a saltmarsh sediment [16]. These findings suggest substantial DMSP cycling activity under anoxic conditions. However, the microbial diversity and metabolic pathways underpinning this process remain entirely uncharacterised.

In addition to the intrinsic importance, DMSP-derived DMS is a substrate for methanogens and sulfate-reducing bacteria (SRB) in anoxic sediments, ultimately contributing to the production of potent greenhouse gases, methane and CO_2_ [3, 17, 18]. Given that saltmarshes -though covering just 0.01% of the Earth’s surface- are hotspots for methane cycling and contribute disproportionally to global emissions, identifying the sources of DMS is crucial for addressing key knowledge gaps in greenhouse gas production within anoxic saltmarsh sediments [19].

To investigate the unstudied potential of anaerobic DMSP degradation as well as the active microorganisms and metabolic pathways involved in DMSP utilisation as a carbon source in anoxic saltmarsh sediments, we employed stable isotope probing (SIP) using isotopically labelled DMSP, complemented by 16S rRNA gene sequencing and metagenomics. Our study also aimed to address key knowledge gaps in the role of DMSP degradation for DMS production and its downstream contribution to methane and CO_2_ productions in anoxic sediments.

## Materials and methods

### Study site and sediment sampling

**Figure 2:**
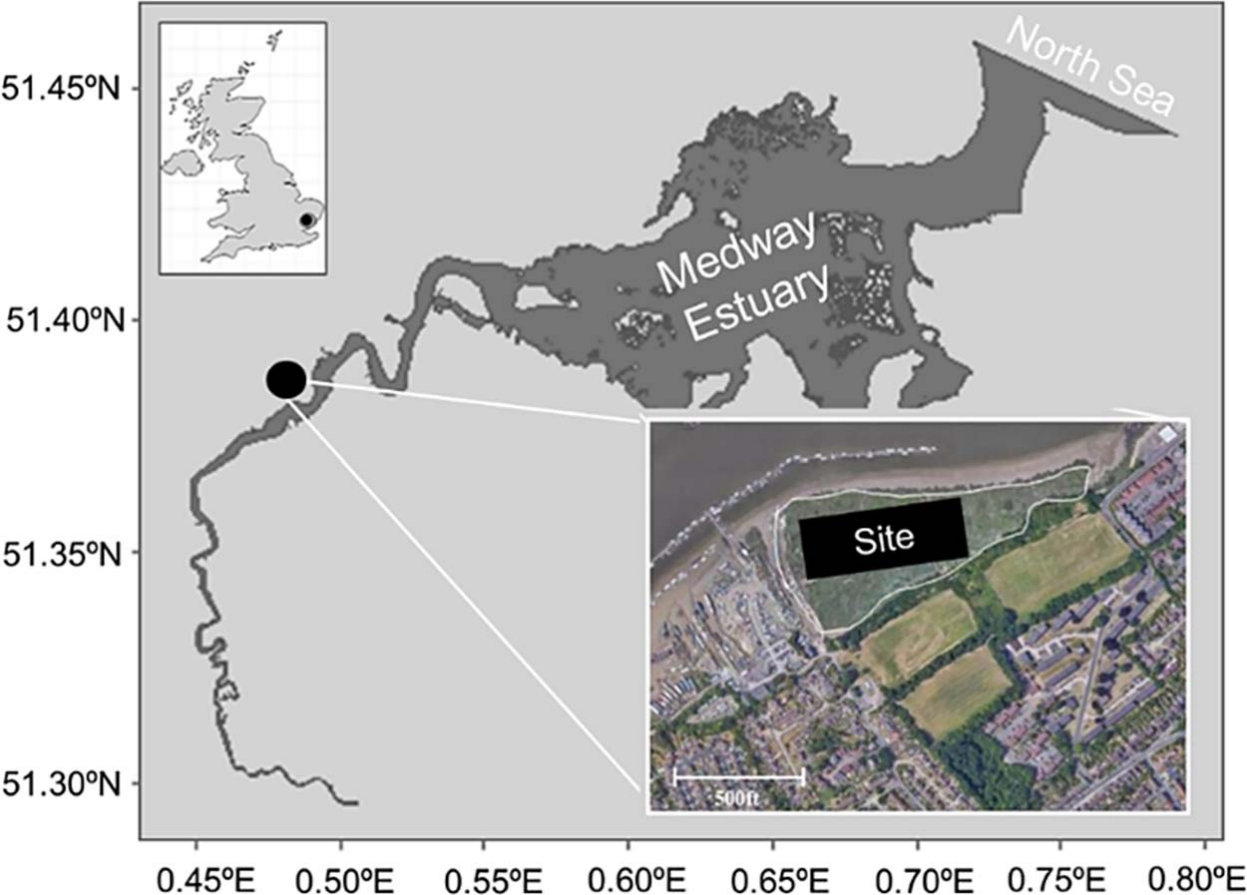
Map of sampling site with coordinates. Black dot represents the brackish (6‰ salinity) saltmarsh sampling site along the Medway estuary. Sampling was carried out at low tide in March 2023.

Sampling took place at saltmarsh on the Medway Estuary, Kent (Southeast England) in March 2023 (Fig. 2). The saltmarsh is a brackish site (~6‰ salinity), which is dominated by *Spartina* spp. Three replicated sediment cores were collected using 3.5 cm Perspex corers at low tide and sealed with gas-tight lids. The average ambient temperature during sampling was 2°C. Sediment cores were transported to Queen Mary University of London (UK) and kept at 4°C in a dark room until further processing.

Sub-samples were collected every 3 cm from one core at each site for in situ measurements of DMSP, methane and CO_2_ as described below. For DMSP measurements, a 3 ml sterile syringe was used to remove sediment from each depth, which was weighed and decanted into a 12 ml gas-tight vial (Labco Exetainer®). Anoxic deionised water with 10 mM NaOH (Sigma-Aldrich®) was added at a 1:1 ratio to liberate DMS. After incubating overnight in the dark, DMS in the headspace was measured. For in situ methane and CO_2_ measurements, 12 ml gas-tight vials were pre-filled with supersaturated NaCl (Sigma-Aldrich®) and sparged with oxygen-free nitrogen (N_2_, British Oxygen Company, BOC, UK). Next, 0.5 ml of sediment was removed every 3 cm from the corer and rapidly decanted into the pre-filled 12 ml vials, which were left in the dark upside down for headspace measurements.

### DMSP, DMS, methane and CO_2_ measurements

Manual headspace measurements were carried out using a gas-tight syringe (Hamilton®, UK), taking 100 μl of headspace. Methane and CO_2_ were measured using a gas chromatograph fitted with a Flame Ionization Detector (FID) and hot-nickel catalytic converter (Agilent Technologies, USA, 6890 N Series). A stainless-steel column (1.83 m × 3.18 mm Ø) packed with Porapak (Q 80/100) was used, with the oven set to 30°C. High-purity nitrogen (14 mL/min) was employed as the carrier gas. The FID was operated at 300°C, with hydrogen and air (zero grade, BOC, Industrial Gases, Guilford, UK) at flow rates of 40 mL/min and 430 mL/min, respectively (7/93% ratio). Methane concentrations were determined from peak areas calibrated against known standards by diluting pure methane (BOC, Guilford, UK). Total methane concentrations included dissolved methane in slurries calculated using atmospheric equilibrium solubility equation [20].

DMS was measured using a gas chromatography (Agilent Technologies, 6890A Series, USA) equipped with a Flame Photometric Detector (FPD). Nitrogen (BOC, UK) was used as the carrier gas. The oven was run at 180°C with a flow rate of 26.7 mL/min. The FPD was operated at 250°C, utilizing hydrogen and air (BOC, UK) at flow rates of 40 mL/min and 60 mL/min, respectively. Standards of gaseous DMS, produced by diluting >99% DMS (Sigma-Aldrich®, USA) ranging from 0.01 mM to 1 mM, were measured daily with two injections per standard. Standards were made anoxic by sparging 50 ml gas-tight bottles with N_2_ (BOC).

DMSP was measured by taking 200 μl of the slurry out of the incubations with a gas-tight syringe (Hamilton®, UK). This was transferred to 2 ml gas-tight vials (Sigma-Aldrich®, USA), which were heated in a water bath at 80°C for 15 minutes without caps. Next, the vials were crimp-sealed, injected with 100 μl (10 M) of NaOH (SigmaAldrich®, ACS reagent), shaken and then stored at room temperature for 2 hours before measuring liberated DMS in the headspace.

### DNA-SIP experiments

Each SIP incubation was set up in an anoxic hood (CV204; Belle Technologies) in a 50 ml gas-tight bottle, which was amended with 2 g of mixed sediment from 6–9 cm depth from each core and 20 ml of 50% Artificial Sea Water (ASW) [21]. Triplicate incubations were amended with 8 μmol/g of either ^13^C-DMSP or ^12^C-DMSP. ^13^C-DMSP was synthesised from acrylic acid-^13^C_3_ and DMS (Sigma-Aldrich) [22]. Three additional incubations were set up with no DMSP as controls. Incubations were kept at room temperature (21°C) in the dark to avoid the photodegradation of DMS [23]. In the DMSP amended incubations, 8 μmol/g of DMSP was added daily until the incubations reached a total of approximately 100 μmol/g carbon assimilation. Incubations were terminated after 12 days, the sediment slurry was decanted into 50 ml centrifuge tubes and centrifuged at 1000 rpm for 4 minutes (ALC laboratory centrifuges PK131R, UK). The liquid was decanted into 10 ml tubes and stored at −20°C for subsequent analyses. The remaining sediment was collected and stored at −20°C for DNA extraction.

### SIP DNA fractionation

Extracted DNA from the ^13^C-DMSP and ^12^C-DMSP SIP incubations was first centrifuged at 44100 r.p.m. using Beckman Optima L-80XP ultracentrifuge with a Vti 65.2 rotor as described before [24]. Following this, gradient fractionation was applied to separate the DNA into heavy and light DNA fragments. The density of each fragment was determined by refractometry using a Reichert AR200 refractometer (Reichert Analytical Instruments).

### DNA extraction and polymerase chain reaction (PCR)

DNA was extracted from 0.25 g of sediment using the DNeasy Powersoil kit (Qiagen, NL), following the manufacturer’s instructions. Extracted DNA was assessed on a 0.8% agarose gel and was quantified using the Qubit dsDNA BR Assay Kit and the Qubit 2.0 Fluorometer (Invitrogen, CA, USA).

The 16S rRNA genes of bacteria and archaea were amplified using OneTaq Quick-Load 2x Master Mix with standard buffer (NEB®, USA) and the universal primer set 515F and 806R, which were equipped with overhang adaptors [25, 26]. PCR amplification was carried out in a final volume of 25 μl with 32 cycles in an iCycler thermal cycler (Bio-Rad®, USA) at an annealing temperature of 55°C. All PCR products were purified using JetSeq Clean beads (1.4x; Meridian Bioscience®, USA) according to the manufacturer’s instructions.

### High-throughput sequencing and processing of the sequences

The cleaned PCR products were incorporated dual indices and Illumina sequencing adapters. Each reaction mixture included 2 μL of purified PCR products, 1 μL of each primer (5 μM), 12.5 μL of 2x Q5 Hot-start Ready mix (NEB, USA), and 8.5 μL of ultra-pure water. The PCR protocol commenced with an initial denaturation at 98°C for 3 minutes, followed by 8 cycles of denaturation at 98°C for 20 seconds, annealing at 55°C for 15 seconds, extension at 72°C for 15 seconds, and a final extension step at 72°C for 5 minutes. All samples were then normalized using the SequalPrep Normalization Plate (96-well) kit (Invitrogen, USA) according to the manufacturer’s instructions. The normalized PCR products were sequenced on the Illumina MiSeq v3 platform (300 bp paired-end, Illumina, USA).

The amplicon sequencing data was analysed using QIIME2 2021.11 on Queen Mary’s Apocrita HPC facility, supported by QMUL Research-IT [27, 28]. Paired-end demultiplexed sequences were processed through the DADA2 pipeline [29]. Primer sequences were removed from the paired-end reads, along with low-quality data below a quality score of 35, and chimeric sequences were filtered out. After the filtering steps, the DADA2 pipeline was used to assign Amplicon Sequence Variants (ASVs) [29]. To perform genus-level analysis, ASVs were grouped into Operational Taxonomic Units (OTUs) with an 99% similarity threshold. Taxonomy was assigned to OTUs using a pre-trained Naïve Bayes classifier acquired from SILVA for 16S rRNA gene.

### Statistical analysis

Principal Coordinate Analysis (PCoA) was carried out using Bray–Curtis dissimilarity index, enabling a visualization of the dissimilarity between groups. To compare the differences between the PCoA groups, a pairwise Permutational Multivariate Analysis of Variance (PERMANOVA; 9999 permutations) was carried out using the “adonis” function from the Vegan package (version 2.4–6) [30]. The “DESeq2” package (version 3.19) was used to estimate changes in taxonomic abundance between treatments. One-way ANOVA was also implemented using R to carry out comparisons between various grouping, followed by a post-hoc Tukey Test to determine significant variables. To analyse the relationships between key variables, the Spearman correlation matrix was computed using R. The “corrplot” package was then used to visualize the correlation matrix.

### Metagenomics analysis

Paired-end (2x150 bp) metagenomic sequencing of the DNA from the original sediment sample, ^13^C-DMSP SIP incubations and the light fraction of the ^12^C-DMSP SIP incubations was carried out using Illumina Nextera sequencing by the Joint Genome Institute (JGI) of the U.S. Department of Energy (DOE). All DNA was at a concentration of 4–40 ng/μL and the absorption ratios (A260/280) ranged from 1.5–2.4 (Nanodrop One, Thermo Scientific, USA).

The metagenomic datasets were analysed by DOE JGI following their well-established pipeline [31]. After filtering contaminants, adapter sequences, and low-quality reads, the reads were assembled using metaSPAdes (pipeline version 4.1.6) and mapped back to contigs with BBMap (version 39.03). The assembled contigs were used for feature prediction, following the IMG Annotation System (IMGAP v5.1.17) pipeline, which incorporates databases such as IMG-NR, SMART, COG, TIGRFAM, SuperFamily, Pfam, and Cath-Funfam to carry out functional annotations using lastal 1256 and HMMER 3.3.2.

Assembled contigs were used to generate metagenome assembled genomes (MAGs) with MetaBAT (version 2.12.1) [32] with a 3000 bp minimum contig cutoff. Genome completeness and contamination were evaluated using CheckM (version 1.0.12) [33] according to the Minimum Information about a Metagenome-Assembled Genome (MIMAG) standards [34]. The Integrated Microbial Genome (IMG) [35] and GTDB-Tk (version 0.2.2) [36] databases were used to determine the taxonomic affiliations. Additionally, a total of 20 genes involved in DMSP and DMS cycling were quantified in the metagenomics sequences and MAGs (Supplementary Material). Hits were discarded if they had <60% amino acid identity to the corresponding ratified proteins using a cut-off value of E ≤ 1e−30 [37]. The final gene counts were normalized to Copies Per Million (CPM). The package “ggplot2” was used to produce a heatmap of the genes in R (Version 3.5.1).

## Results

### DMSP, methane and CO_2_ concentrations in natural saltmarsh sediment

**Figure 3:**
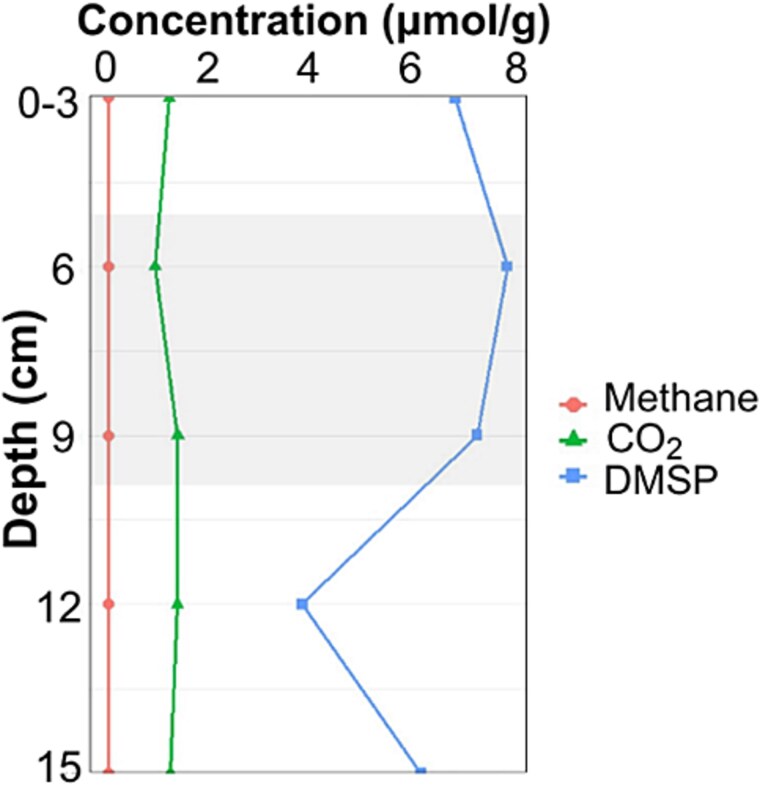
Sediment depth profile of DMSP, methane and CO_2_ as measured per gram of wet sediment. Depths from 0–15 cm were measured every 3 cm (biological replicate n = 1; technical replicate n = 1). Grey area indicates sediment that was collected for SIP incubation.

Anoxic sediment samples from the Medway Saltmarshes were collected to quantify DMSP, methane and CO_2_. Measurements of sediment samples every 3 cm to the depth of 15 cm show endogenous DMSP concentrations at every sediment layer. The highest DMSP concentration was measured at 7.7 μmol/g wet sediment in samples from 3 to 9 cm. The average DMSP, methane and CO_2_ concentrations were 6.1 ± 3.8 μmol/g wet sediment, 0.01 ± 0.01 μmol/g wet sediment and 1.3 ± 0.3 μmol/g wet sediment, respectively (Fig. 3).

### Candidate microorganisms and pathways producing DMSP in anoxic saltmarsh sediment

**Figure 4:**
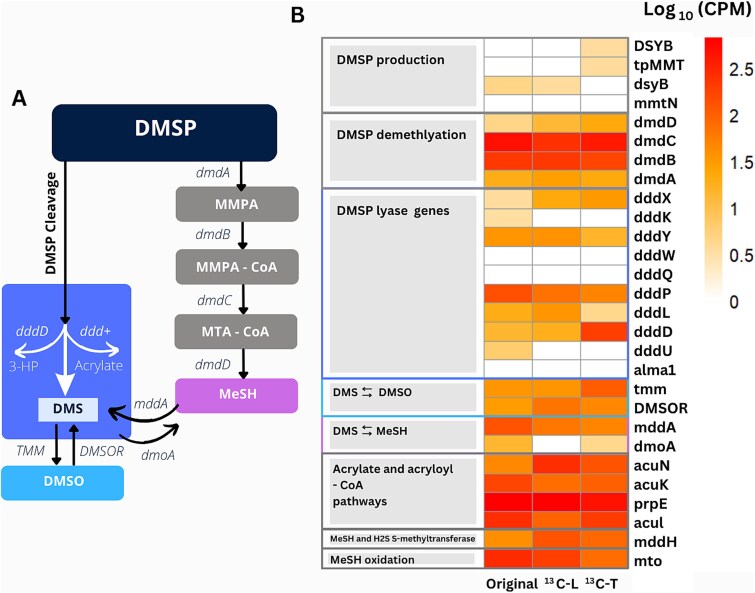
A) the cleavage and demethylation pathways of DMSP degradation. Genes indicated were searched in the metagenomic datasets from the original sediment sample, ^13^C-light SIP DNA fraction and ^13^C-Total DNA. B) Heatmap of genes involved in DMSP cycling in the original sediment sample, ^13^C-light SIP DNA fraction and in the ^13^C-Total DNA. Scale shows log of gene copies per million (CPM).

A metagenome (MG) dataset from the original sediment sample (6–9 cm depth) was quality filtered and a total of 184 882 470 reads were assembled into contigs. These were then analysed for the genetic potential to synthesise DMSP via known pathways and enzymes. *dsyB* with a relative abundance of 0.0004% of the MG sequences, was identified as the only DMSP synthesis gene detected in the original sediment samples (Fig. 4). DsyB catalyses the key step of bacterial DMSP synthesis via three methionine transamination pathways [1]. *dsyB* sequences in the MG datasets were 72% identical at the amino acid level to DsyB from *Roseovarius*, consistent with marine *Roseobacter* group being important DMSP producers [15]. Other genes for DMSP production in algae (DSYB and TpMMT) and in bacteria (*mmtN*) were not detected in the original sediment MG (Fig. 3) [38]. Furthermore, 32 high and medium quality MAGs were recovered using the assembled contigs from the original sediment MG dataset. None of these MAGs were associated with *Roseovarius*, nor did they contain the *dsyB* or *mmtN* genes (Supplementary Fig. S1).

### DMSP uptake in SIP incubations

**Figure 5:**
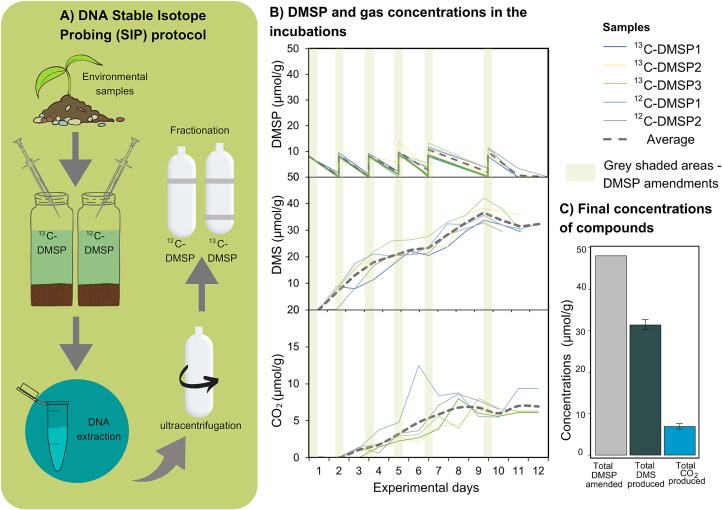
A) DNA-SIP protocol including incubation with isotopically-labelled and unlabelled DMSP, DNA extraction, ultracentrifugation and the fractionation of heavy and light DNA. B) DMSP, DMS and CO_2_ concentrations in the SIP incubations with labelled and unlabelled DMSP. A total of 48 μmol/g DMSP were injected over 12 days, which led to the production of DMS and CO_2_. C) Total DMSP concentration amended to the incubations and average concentrations of DMS and CO_2_ produced by the end of the incubation period.

Known aerobic DMSP-degrading bacteria use the propionate moiety of DMSP for carbon assimilation and produce DMS or MeSH via lysis or methylation pathway, respectively. Therefore, we used ^13^C-acrylate with ^12^C-DMS to synthesize isotopically labelled DMSP and incubate sediment samples. We observed DMSP degradation without a lag phase. In the first 24 hours, 7.51 ± 0.72 μmol/g wet sediment DMSP was degraded and there was simultaneous production of DMS (6.96 ± 3.34 μmol/g wet sediment). Over 12 days of incubation, a total of 48 μmol DMSP/g wet sediment was amended and degraded. A total of 34 (±3.2) μmol DMS/g wet sediment was produced while no MeSH was detected (Fig. 5). There was no significant difference in DMSP degradation or DMS production between the incubations amended with ^13^C- or ^12^C-DMSP (P > 0.05). The controls with no DMSP added, produced <0.01 μmol/g DMSP, DMS and methane, while 7.3 (±2.2) μmol/g of CO_2_ was measured after 12 days. There was a significant correlation between DMS and CO_2_ production with DMSP degradation (P < 0.01).

### Identification of active microbes and pathways underpinning DMSP use as a carbon source

DNA was extracted from the incubations on day 12 after 48 μmol DMSP/g wet sediment was consumed. Following fractionation, taxonomic characterisation was carried out via the 16S rRNA gene and metagenomics sequencing.

**Figure 6:**
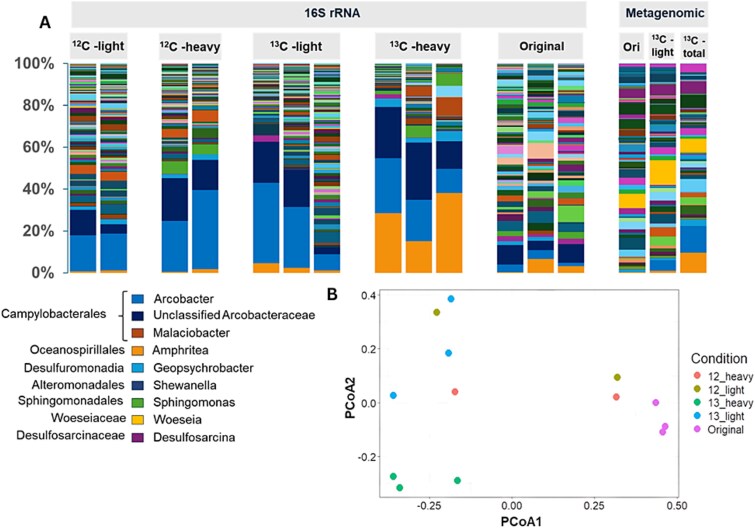
16S rRNA gene and metagenomics sequencing results. A) Relative abundance of the 16S rRNA gene sequences at the genus-level in the original sediment sample, ^12^C-heavy, ^13^C-heavy and ^13^C-light DNA following DNA-SIP fractionation and the taxonomic analysis of the metagenomics sequences from the original sediment, ^13^C-light DNA and ^13^C-Total DNA. B) PCoA of the 16S rRNA gene sequence data demonstrating the difference in community composition between the ^12^C-and ^13^C-labelled heavy and light DNA fractions.

A total of 434 070 quality-filtered, chimera-free sequences were obtained from heavy and light DNA fractions from all SIP incubations. Bacteria dominated the community across all the samples (97.6% ±1.7 of the total 16S rRNA gene sequences), while archaeal communities comprised less than 5% in every fraction and there was no significant difference in archaeal relative abundance between fractions (P > 0.05; Fig. 6). The most abundant family across all heavy and light DNA fractions was *Arcobacteraceae* from the order *Campylobacterales*. This family was also the most dominant taxon in ^13^C-Light and ^13^C-Total metagenomes at 39% and 41% relative abundances, respectively. However, there was no significant increase in the relative abundance of this family in the ^13^C-Heavy compared to light fractions according to the 16S rRNA gene sequencing (P > 0.05). Strikingly, the family *Nitrincolaceae* was significantly enriched in the ^13^C-Heavy DNA to 27.2 ± 9.1% from 2 ± 1.3% in ^13^C-Light, ^12^C-Heavy and ^12^C-Light DNA fractions (P < 0.05; Fig. 5). More specifically, *Amphritea* within this family increased significantly (P < 0.05) to 26.9 ± 9.1% in the ^13^C-Heavy DNA compared to other fractions. Similarly, the relative abundance of *Amphritea* increased significantly in the metagenome from the ^13^C-Total DNA (7.21%) when compared to the ^13^C-Light fraction (1.92%) and the original sample (0.43%). Overall, these results suggest *Amphritea* to be the active population assimilating carbon from DMSP.

*Geopsychrobacter* also significantly increased in the ^13^C-Heavy DNA to 3.9 ± 1% of the 16S rRNA gene sequences compared to all other fragments (<0.9%; P < 0.05, Fig. 6), suggesting this genus likely used DMSP or the metabolites of DMSP degradation as a carbon source.

### Potential pathways of anaerobic DMSP degradation

Metagenome datasets from ^13^C-light DNA fraction and ^13^C-total DNA were quality filtered. A total of 20 908 239 and 14 045 719 reads were assembled into contigs and analysed to elucidate the potential DMSP degradation pathways in anoxic sediments (Fig. 4). MAGs recovered from the metagenome datasets were also searched for 28 genes involved in DMSP and DMS cycling. While the DMSP demethylation (*dmdA*) gene did not enrich in ^13^C-DMSP incubations, the relative abundance of the DMSP lyase gene, *dddD*, showed a significant increase in the ^13^C-Total metagenome. It increased 18-fold in the ^13^C-Total metagenome (0.018%) compared to both the ^13^C-Light fraction (0.001%) and the original sediment (0.001%; Fig. 4). While we did not recover any MAGs affiliated with *Amphritea*, 89% of the *dddD* genes detected in the ^13^C-Total metagenome were closely related to *Amphritea atlantica* DSM 18887 (97% amino acid identity). *dddP* was also found in the original sediment, ^13^C-Light and ^13^C-Total DNA, while *dddQ*, *dddW* and Alma1 were at very low abundances or absent in all samples. Three MAGs from the original sediment identified as *Gammaproteobacteria*, *Myxococcota* and *Rhodobacteraceae* contained the *dddP* gene, whereas only one MAG (*Acidimicrobiales*) was found to have *dddD* (Supplementary Fig. S1). Similarly, *dddX* and *dddQ* were found in one MAG each (*Ilumatobacter* and *Gammaproteobacteria*, respectively; Supplementary Fig. S1). Among the four MAGs recovered from the ^13^C-Light DNA, three contained a distinct *ddd* gene (*dddD*, *dddX* and *dddP*), whereas MAGs from the ^13^C-Total DNA lacked genes associated with aerobic DMSP lyase metabolism (Supplementary Fig. S1).

To gain insight into the fate of DMS in our incubations, we searched the genes involved in DMS biotransformations in the MG datasets. The *tmm* gene, which facilitates the oxidation of DMS to DMSO, was significantly enriched in the ^13^C-Total DNA (0.01%) compared to both the ^13^C-Light fraction (0.003%) and the original sediment (0.003%; Fig. 4). The *tmm* sequences found in ^13^C-Total DNA were primarily associated with *Amphritea pacifica* RP18W (95% amino acid identity). Meanwhile, *dmoA*, which expresses the enzyme catalysing the conversion of DMS to MeSH, was absent in the ^13^C-Light fraction but detected in both the ^13^C-Total (0.0004%) and the original MG datasets (0.001%). We also searched for the genes catalysing MeSH transformations. The *mto* gene that oxidises MeSH was relatively abundant in the original MG (0.028%) but did not increase in other MG datasets, while the *mddH* gene (methylating MeSH to DMS) increased significantly to 0.0128% in the ^13^C-Light fraction from 0.0039% from the original MG [39, 40]. A total of nine high and medium quality MAGs were recovered from the assembled contigs of the SIP MG datasets (Supplementary Fig. S1). The *mto* gene was found in one MAG (Gammaproteobacteria_UBA9214) while two MAGs contained the *mddH* gene (*Anaerotignaceae*_UBA8514 and *Desulfuromonadales*_BM103).

### Environmental distribution of *Amphritea*

**Figure 7:**
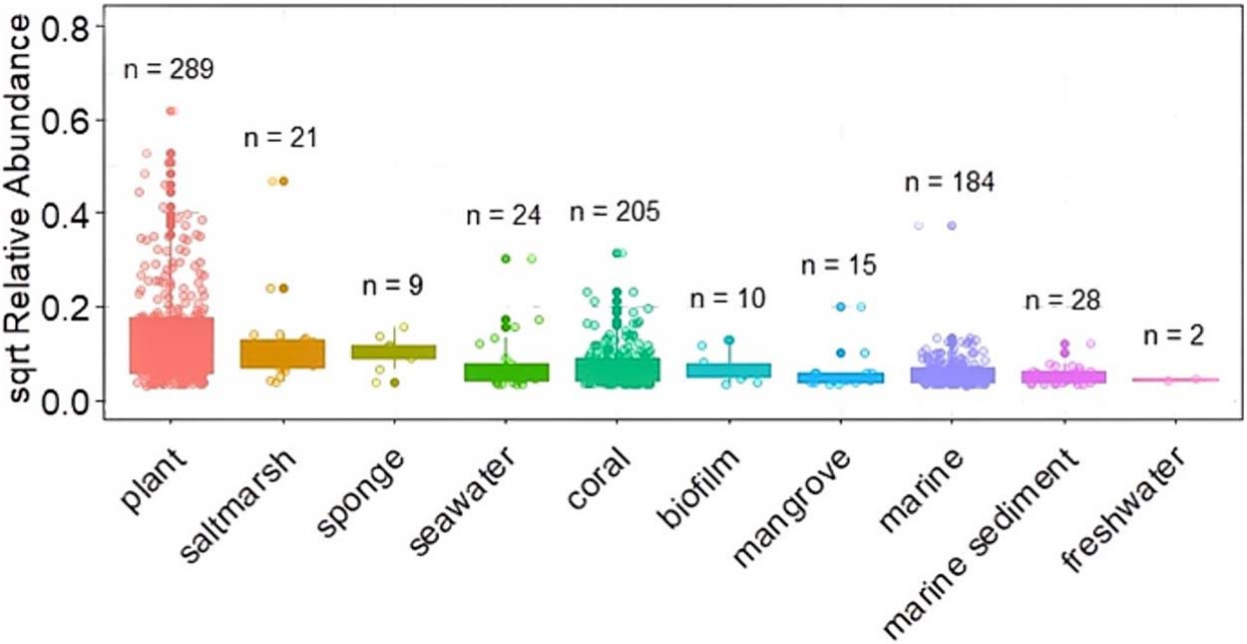
Relative abundance of publicly available *Amphritea pacifica strain ZJ14W* derived from 16S rRNA amplicon sequences from global datasets. Data recovered from IMNGS.

To understand the global importance of the genus *Amphritea*, we assessed how widespread *Amphritea* species were across various ecosystems using publicly available databases. The nucleotide sequence of *Amphritea* obtained from 16S rRNA gene sequencing was analysed using BLAST, which identified the closest matching sequence in the nucleotide database as *Amphritea pacifica strain ZJ14W*. In total, 788 publicly available *Amphritea* OTU were recovered from databases, showing the distribution of this genus across different habitats including saltmarshes and marine sediments (Fig. 7).

## Discussion

### DMSP cycling in anoxic sediments

This study represents the first analysis of in situ DMSP concentrations in anoxic sediments and provides novel insights into the microbial populations and pathways involved in anaerobic DMSP biotransformations, a process that has remained unexplored until now.

The high DMSP levels (6.1 μmol/g ± 3.8) observed in anoxic saltmarsh sediments add to the research identifying saltmarshes as hotspots of DMSP cycling [15, 41]. Notably, the in situ DMSP concentrations in our samples are considerably higher than those reported in the earlier studies, which may be attributed to differences in sampling location. Our samples were collected in close proximity to *Spartina* plant roots, which likely contributed to the elevated DMSP levels. *Spartina spp*., a dominant saltmarsh plant, has been proposed to account for approximately 10% of global atmospheric DMS emissions, highlighting the high DMSP productivity of saltmarshes per unit area compared to oceans [1]. DMSP production in saltmarshes is attributed not only to *Spartina spp*. but also to various bacteria and algae that produce DMSP as an osmoprotectant [1, 15]. Metagenomics data showed that bacterial *dsyB* associated with *Roseovarius* were the only DMSP synthesis gene detected in our samples. Genes associated with algal production or bacterial methionine methylation were not detected, overall implying that bacterial methionine transamination via *dsyB* is the dominant pathway for DMSP synthesis in our saltmarsh sediments. Nevertheless, the presence of novel DMSP synthesis genes in diverse bacteria within the anoxic sediments remains a possibility, as previously proposed [15].

In the DMSP-amended sediment incubations, we observed immediate DMSP breakdown and DMS production at significant concentrations, clearly suggesting the presence of DMSP-catabolising microbes in anoxic saltmarsh sediments. To date, only one other study has indicated that DMSP is degraded to DMS in anoxic sediment, where both DMS and MeSH were measured [42]. Stoichiometrically, one mole of DMSP can yield one mole of DMS, showing 68.6% (±4.2%) of DMSP was converted to DMS in our incubations. A previous study on aerobic saltmarsh sediments has shown a similar conversion efficiency of approximately 72% [41], which suggests that DMSP lysis, producing DMS as a product, operates at comparable rates under both aerobic and anaerobic conditions. The prevalence of the DMSP lysis pathway may be due to the DMSP concentrations in sediment since high concentrations (10 μM and above) of DMSP disproportionately increase the lysis pathways [43]. Here, the concentration in the sediment and in the incubations is above this threshold, which may explain the dominance of the lysis route that produces DMS.

It is plausible that, during our incubations, DMS was produced in higher concentrations than those measured but underwent concurrent degradation. Indeed, light fractions from the SIP incubations had increased relative abundance of *mddH* that produces DMS from MeSH. DMS was likely degraded by SRB as we detected sulfate-reducing genera such as *Desulfosarcinaceae* in both original sediment and SIP incubations, suggesting SRB activity. This could also explain the high CO_2_ levels in the incubations. Also, we did not detect methane, a DMS degradation product, likely due to short incubation times (12 days) compared to previous studies, where methane production was observed after around 12 days [18, 44]. DMS may also have been oxidised to DMSO via *tmm* or degraded to MeSH via *dmoA* as these genes were detected in the original and ^13^C-total MG datasets. Nevertheless, high conversion efficiencies of DMSP to DMS has far-reaching implications for climate systems, as DMS can be further metabolized to produce methane and CO₂, both potent greenhouse gases in anoxic environments [18, 45]. Coastal ecosystems such as saltmarshes are responsible for 60% of total marine methane emissions, and the contribution of DMSP and its degradation products may play a critical, yet previously unrecognized, role in these emissions [19].

### Active microorganisms and pathways underlying anaerobic DMSP breakdown

We identified the active microorganisms that used DMSP as a carbon source in anoxic saltmarsh sediments using partially labelled DMSP in a SIP experiment. The significant proliferation of *Amphritea* from the order *Oceanospirillales* in the ^13^C-Heavy fractions indicate this genus as the active DMSP-carbon assimilators in the saltmarsh sediment studied. *Amphritea* have previously been found to actively degrade DMSP in aerobic ecosystems via either the demethylation pathway (*dmd*) or the lysis pathway (*ddd+*) [12, 41]. In our samples, we found that the *dddD* genes, which proliferated in the SIP incubations, were associated with *Amphritea*, implying that this genus employed the lysis pathway for DMSP degradation. We did not find these functional genes associated with *Geopsychrobacter* or *Arcobacteraceae*. Similarly, in our previous work, we carried out SIP on aerobic saltmarsh sediments and showed that *Amphritea* species likely used *dddD* for DMSP lysis, resulting in the liberation of DMS and 3-HP [1]. However, it should be noted that, our study is based on DNA analysis, therefore, further investigation using RNA or protein-based approaches is required to confirm the metabolic pathways involved.

Interestingly, our current study revealed that the observed conversion efficiency of DMSP to DMS under anoxic conditions (~68%) is comparable to aerobic environments (72%), strongly suggesting that *Amphritea* has a similar efficiency in anoxic sediments. It is likely that *Amphritea* degrade DMSP as a key carbon source as supported by its common presence in marine and coastal environments, particularly in regions characterized by high DMSP production, such as saltmarsh sediments. This genus has also been frequently detected in microbiomes associated with DMSP-producing organisms, including macroalgae, phytoplankton, and saltmarsh plants like *Spartina* spp. [1, 41]. Its distribution in marine ecosystems and metabolic versatility suggest that *Amphritea* is a globally important DMSP-degrader, capable of adapting its metabolic strategies to varying redox conditions. Notably, we previously found other *Oceanospirillales* taxa in aerobic sediments to contain DMSP cycling proteins. For instance, *Marinobacterium*, *Oceanospirillum*, and *Marinomonas* possess the *dddD* (DMSP lysis) gene and, to a lesser extent, the demethylation gene *dmdA* [41]. This further implicates *Oceanospirillales* to play an important role in both aerobic and anaerobic DMSP cycling.

One of the most notable novel enrichments observed in the ^13^C-Heavy DNA was the genus *Geopsychrobacter*, which suggests that these bacteria potentially used DMSP as a carbon source. *Geopsychrobacter* species are versatile in their substrate utilisation, being capable of metabolising a wide range of organic compounds, including acetate, amino acids, long-chain fatty acids and aromatic compounds [46]. It is also possible that *Geopsychrobacter* species were labelled due to cross-feeding on metabolites generated during DMSP catabolism by the direct assimilators. However, our study could not confirm this, as we conducted a single time-point SIP experiment. Members of *Geopsychrobacter* are also known for their capacity to harvest electricity through the oxidation of organic compounds, producing CO₂ as a byproduct [46]. This metabolic trait could explain the observed increase in CO₂ levels in the DMSP-incubated sediments. This is further supported by the significant correlation between CO₂ production and DMSP degradation during the incubations, implicating a direct link between DMSP metabolism and CO₂ production in anoxic sediments. Further study of the DMSP metabolism of this genus would, therefore, be particularly noteworthy.

We found *Arcobacteraceae* to be the most abundant family across all DNA fractions. However, this family was not significantly enriched in the ^13^C-Heavy fractions, which could be due to its high GC content, hindering the buoyant density shift necessary for distinguishing heavy isotopic fractions [24]. Nevertheless, the *Arcobacter nitrofigilis* genome contains a *dddY*-like gene and this species has been shown to produce DMS from DMSP [47]. Therefore, it remains plausible that members of *Arcobacteraceae* degraded DMSP in our samples. Alternatively, *Arcobacteraceae* might have respired DMSP-derived acrylate or used DMSP as a sulfur source, which likely substantially increased its abundance in both ^12^C- and ^13^C-DMSP incubations whilst keeping their DNA carbon isotopically unlabelled. *Arcobacteraceae* were shown to cycle sulfur and grow mixotrophically previously [38]. In support of this, two *Arcobacteraceae* MAGs from the original sediment contained genes expressing proteins involved in sulfur cycling such as *dsbC* and *soxY*. *Arcobacteraceae* may therefore be implicated in the anaerobic cycling of DMSP sulfur, however, further research is required to confirm this.

This is the first study revealing the microorganisms involved in DMSP cycling in an anoxic sediment, offering a novel perspective on the contribution of these ecosystems to global carbon and sulfur cycles, as well as to the production of the climate-active gas DMS. The significant enrichment of *Amphritea* and the *dddD* gene in ^13^C-Heavy SIP fractions strongly implicates this genus with the DMSP lysis pathway as key players in DMSP-derived carbon assimilation under anoxic conditions. The presence of *Amphritea* across diverse marine and coastal environments raises intriguing questions about its broader ecological role beyond DMSP cycling. Its apparent capacity for anaerobic DMSP degradation positions it as a key intermediary linking the organosulfur cycle to methane and CO_2_ production in such environments. Validating the anaerobic metabolic capabilities of *Amphritea* and the broader *Oceanospirillales* order through metatranscriptomic and metaprotemics analyses as well as pure culture studies would not only expand the known metabolic repertoire of these species but also show the underexplored pathways of DMSP degradation in anoxic environments. Moreover, the possibility that different DMSP-utilizing microbes and degradation pathways are active in other anoxic settings, such as estuarine or marine sediments, warrants further investigation.

## Supplementary Material

Supplementary_materials_ycaf180

## Data Availability

All sequence data produced in this study are publicly available. The 16S rRNA gene sequences are deposited at the National Center for Biotechnology Information (NCBI) Read Archive (PRJNA1292712). Metagenomics datasets are available at JGI GOLD database (Project IDs: Ga0669903, Ga0654325 and Ga0654326).
