# Supplementary material for: Active microorganisms and potential metabolic pathways mediating anaerobic degradation of DMSP in anoxic saltmarsh sediment
Source: ISME Commun. 2025 Sep 9;5(1):ycaf180. doi: 10.1093/ismeco/ycaf180 (PMC12596276; doi:10.1093/ismeco/ycaf180)
Supplement: Supplementary_materials_ycaf180 [file supplementary_materials_ycaf180.pdf]

Active microorganisms and potential metabolic pathways mediating anaerobic degradation of DMSP in anoxic saltmarsh sediment

Susan E. G. Hawthorne[1], Stephania L. Tsola[1], Ornella Carrión[2],
Jonathan D. Todd[2] and Özge Eyice[1,3]*

Supplementary Material

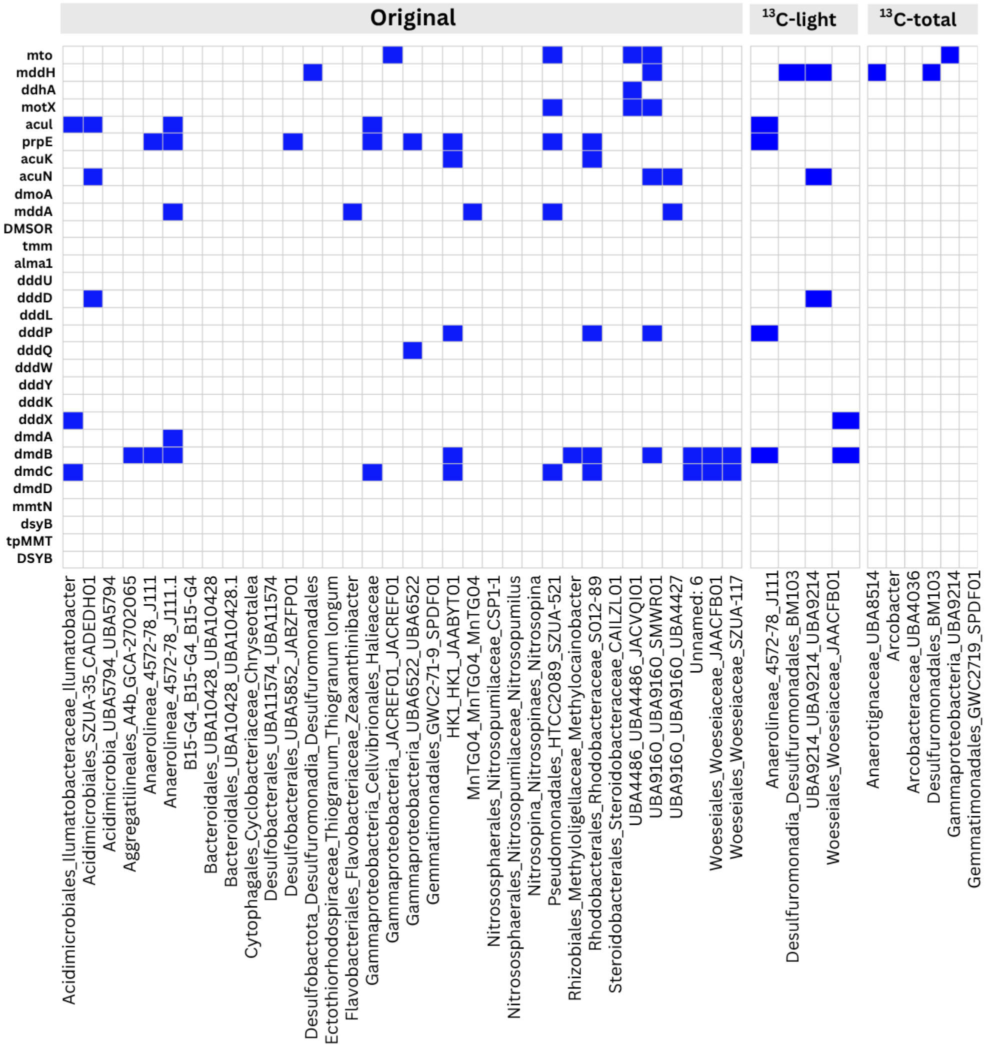

Supplementary Figure 1. MAGs recovered from the original sediment metagenomic dataset and distribution of the genes involved in the cycling of DMSP and its metabolites.
